# Book Review: East Asian Perspectives on Silence in English Language Education

**DOI:** 10.3389/fpsyg.2021.721438

**Published:** 2021-06-25

**Authors:** Ying Huo

**Affiliations:** Department of Foreign Language, Northwest University of Political Science and Law, Xi'an, China

**Keywords:** English language education, education, learner silence, East Asian perspectives, English language

Learner silence in EFL/ESL classrooms is an interactional and interpersonal resource, intentionally or subconsciously drawn on to indicate the psychological or linguistic difficulties learners face. Besides, it can be construed as a signal of the socio-cultural viewpoints that can function as frameworks for language learning. “Silence is a pedagogical issue that touches all who teach.” (p. 3). Therefore, it is documented that learner silence is an intricate and multidimensional phenomenon that can emerge from a wide range of sources and thus can challenge clear generalizations. Nonetheless, its instrumental role in language classrooms has not been fully investigated. To address this complexity, *East Asian Perspectives on Silence in English Language Education*, edited by Jim King and Seiko Harumi, deepens our nascent awareness and understanding of the silent episodes that take place within East Asian language classrooms. I consider this compendium an opportune collection that is in line with the scope of the Research Topic, entitled *The Role of Teacher Interpersonal Variables in Students' Academic Engagement, Success, and Motivation*, co-guest-edited by Ali Derakhshan, Reza Pishghadam, and Anna Mystkowska-Wiertelak. The main impetus for collecting such an unprecedented volume is to unravel the hidden dimensions of learner silence and challenge the stereotype of the reticent, passive East Asian learners by providing contextually rich data.

Following the foreword by *Peter MacIntyre*, the book comprises nine chapters. The editors commence the volume by justifying the fairly underrepresented issue of silence in EFL/ESL classes, enumerate the main objectives of the book, sketch the historical background of silence-related studies since the 1980s, illuminate a wide range of conceptualizations, foreground some fundamental questions by accentuating the crucial role of contextual factors, and problematize the methodological challenges to collect data for issues surrounding silence. Utilizing phenomenological case studies through the lens of interpretive discourse analysis, in Chapter 2, *Dat Bao* probes into task complexity, task orientation, modes of learning, and learner silence. The author collected data through semi-structured interviews from 10 postgraduate students coming from different East Asian backgrounds to an Australian university. This study enlightens us by unpacking the interactive nature of learners' speech dynamics, task modes, learners' choice of verbal responses, and silence, reflected through the metacognitive process of self-revelation.

The main focus of *Seiko Harumi*'s chapter is to investigate the role of silence as an interactional repertoire through involvement in didactic turn-taking interactions and L2 output that occur among Japanese EFL students. Her study uses open-ended questionnaires to find out how teachers react to instances of silence. Her findings illustrated that some teachers felt discomfort during the episodes of silence. Besides, this chapter reports on the 8 h of classroom interaction to delve deeply into student silence, teacher talk, and wait time, resulting in some pedagogical strategies employed by Japanese students.

Observing learner reticence longitudinally from a psychological and complexity vantage point, *Jim King, Tomoko Yashima, Simon Humphries, Scott Aubrey, and Maiko Ikeda* scrutinized the role of silence among Japanese universities in English as a medium of instruction by focusing on the learners' anxiety coping strategies, interpersonal dynamics, and social cooperation. Their findings revealed that fundamental elements that instigate learner silence pertain to learners' anxiety in the use of English, topic choice, task demands, and their social inhibition.

Like Chapter 4, in Chapter 5 *Kate Maher* examines the role of silence and anxiety in the Japanese context based on the postulations that emerged from cognitive-behavioral theory (CBT). Focusing on one single participant, the author aims to sensitize the learner's awareness of how aversive emotions and thoughts can make her reticent in the class. The author concludes that confidence to attend oral participation can be enhanced if students are cognizant of their negative cycles and strategically set goals. In Chapter 6, *Michael Karas and Farahnaz Faez* explore the effect of silence in the communicative language teaching (CLT) class from the viewpoints of Chinese teachers in Canada, by taking into account cognitive, interactive, and sociocultural vantage points. The teachers' highly positive attitudes toward the utilization of silence and its role in L2 learning highlight that different strategies can be used to help reticent students in CLT classrooms.

Unlike Chapter 6 that focuses on the role of silence based on teachers' perceptions, Chapter 7, written by *Simon Humphries, Nobuhiko Akamatsu, Takako Tanaka*, and *Anne Burns*, concentrates on students' perceptions. They scrutinize how Japanese students construe their ability to speak English during different classroom activities. The authors conclude that confidence, motivation, anxiety, and classroom support play vital in oral participation. Utilizing psychological orientation, in Chapter 8, *Jian-E. Peng* reveals the intricate interplay between levels of oral participation, willingness to communicate, situated classroom communication, and silence within Chinse EFL classrooms. The final chapter, by *Amy B. M. Tsui and Rintaro Imafuku*, recapitulates the findings of the previous chapters, highlighting the complexity and dynamicity of L2 student and teacher reticence and calls for more longitudinal studies on the dynamic interrelationship of factors that enhance oral participation in educational settings.

I enjoy reading this compendium with great enthusiasm. This provocative volume offers many merits, some of which are as follows. First, this volume takes advantage of cognitive, cultural, interactional, and psychological, and sociocultural viewpoints by incorporating empirical studies that have been informed by a wide range of innovative theoretical conceptualizations and methodological approaches such as interpretive case study, conversational analysis, longitudinal intervention study, cognition study, etc. Second, at the end of each chapter, there are some “self-reflection/discussion questions” and “recommended readings” which pique the interest of those who are willing to deepen their understanding of the complex role of silence. However, had the editors included studies from other East Asian countries, we would have had a better understanding of the dynamic and complex nature of silence in other EFL/ESL contexts. Taken together, I strongly suggest this timely volume to teachers, researchers, and practitioners who believe that silence plays an essential role in students' engagement in L2 learning.

## Author Contributions

The author confirms being the sole contributor of this work and has approved it for publication.

## Conflict of Interest

The author declares that the research was conducted in the absence of any commercial or financial relationships that could be construed as a potential conflict of interest.

